# The association between airway eosinophilic inflammation and IL-33 in stable non-atopic COPD

**DOI:** 10.1186/s12931-018-0807-y

**Published:** 2018-06-01

**Authors:** Damian Tworek, Sebastian Majewski, Karolina Szewczyk, Justyna Kiszałkiewicz, Zofia Kurmanowska, Paweł Górski, Ewa Brzeziańska-Lasota, Piotr Kuna, Adam Antczak

**Affiliations:** 1 Department of General and Oncological Pulmonology, Medical University of Lodz, Kopcinskiego 22, 90-153 Lodz, Poland; 2 Department of Pulmonology and Allergy, Medical University of Lodz, Kopcinskiego 22, 90-153 Lodz, Poland; 3 Department of Biomedicine and Genetics, Medical University of Lodz, Pomorska 251, 92-213 Lodz, Poland; 4 Department of Internal Medicine, Asthma and Allergy, Medical University of Lodz, Kopcinskiego 22, 90-153 Lodz, Poland

**Keywords:** IL-33, Eosinophils, COPD

## Abstract

**Background:**

Interleukin(IL)-33 is an epithelial alarmin important for eosinophil maturation, activation and survival. The aim of this study was to examine the association between IL-33, its receptor expression and airway eosinophilic inflammation in non-atopic COPD.

**Methods:**

IL-33 concentrations were measured in exhaled breath condensate (EBC) collected from healthy non-smokers, asthmatics and non-atopic COPD subjects using ELISA. Serum and sputum samples were collected from healthy non-smokers, healthy smokers and non-atopic COPD patients. Based on sputum eosinophil count, COPD subjects were divided into subgroups with airway eosinophilic inflammation (sputum eosinophils > 3%) or without (sputum eosinophils ≤3%). IL-33 and soluble form of IL-33 receptor (sST2) protein concentrations were measured in serum and sputum supernatants using ELISA. ST2 mRNA expression was measured in peripheral mononuclear cells and sputum cells by qPCR. Hemopoietic progenitor cells (HPC) expressing ST2 and intracellular IL-5 were enumerated in blood and induced sputum by means of flow cytometry.

**Results:**

IL-33 levels in EBC were increased in COPD patients to a similar extent as in asthma and correlated with blood eosinophil count. Furthermore, serum and sputum IL-33 levels were higher in COPD subjects with sputum eosinophilia than in those with a sputum eosinophil count ≤3% (*p* < 0.001 for both). ST2 mRNA was overexpressed in sputum cells obtained from COPD patients with airway eosinophilic inflammation compared to those without sputum eosinophilia (*p* < 0.01). Similarly, ST2 + IL-5+ HPC numbers were increased in the sputum of COPD patients with airway eosinophilia (*p* < 0.001).

**Conclusions:**

Our results indicate that IL-33 is involved in the development of eosinophilic airway inflammation in non-atopic COPD patients.

**Electronic supplementary material:**

The online version of this article (10.1186/s12931-018-0807-y) contains supplementary material, which is available to authorized users.

## Background

Chronic Obstructive Pulmonary Disease (COPD) is one the most common chronic diseases in adults, and one of the main causes of morbidity and mortality [1]. The prevalence of COPD at Global Initiative for Chronic Obstructive Lung Disease (GOLD) stage 2 or higher has been estimated at 10.1% overall, although the precise figure varies according to the region [2]. A growing body of evidence indicates that eosinophilic COPD is a distinct phenotype of the disease, and one that is characterized by a higher risk of exacerbations but better response to treatment with inhaled corticosteroids [3, 4]. COPD subjects have higher percentages of eosinophils in sputum and higher concentrations of eosinophil cationic protein than healthy controls, and their airway eosinophil numbers further increase during disease exacerbation [5, 6]. Data from the ECLIPSE (Evaluation of COPD Longitudinally to Identify Predictive Surrogate End-points) study showed that over three years of observation, 37.4% of COPD patients demonstrated a persistent blood eosinophil count greater than or equal to 2% [7]. Non-allergic COPD patients with blood eosinophil counts > 250 cells/μL are characterized by higher airway eosinophil numbers, higher sputum interleukin(IL)-5 and haptoglobin levels, greater CCL20 and CCL24 concentrations in bronchoalveolar lavage (BAL) and more pronounced airway remodeling [8].

IL-33 is one of the epithelial alarmins generated in response to danger signals, and is primarily produced by epithelial cells [9]. Recent evidence has demonstrated that IL-33 precedes IL-5 in regulating eosinophil commitment and is required for eosinophil homeostasis [10]. One such result of IL-33 activity is that activates and augments the maturation of hemopoietic progenitor cells (HPC). HPC are considered to be innate effector cells in allergic asthma [11, 12]: They produce an abundance of pro-inflammatory cytokines and differentiate into mature eosinophils in the process of “*in situ hemopoiesis*” at sites of inflammation [13].

The aim of the present study was to determine the presence of an association between IL-33 level, the expression of its receptor and the degree of eosinophilic inflammation in non-atopic COPD.

## Methods

### Subjects

Sixteen non-smoking healthy subjects, 23 non-smoking asthmatics and 25 non-atopic COPD subjects (smokers and ex-smokers with a history of at least 10 pack-years) were included in the first part of the study assessing the association between IL-33 in breath condensate (EBC) with blood eosinophilia (EBC study). Forty patients with COPD (smokers and ex-smokers with a history of at least 10 pack-years), 20 smokers (with a history of at least 10 pack-years) without COPD and 20 healthy non-smokers were included in the second phase examining the association between IL-33 and sputum eosinophilia (Sputum study). COPD was diagnosed according to GOLD criteria by demonstrating irreversible bronchoconstriction following inhalation of salbutamol (www.goldcopd.org). Asthma was diagnosed according to Global Initiative for Asthma (GINA) criteria based on the history of typical respiratory symptoms and positive result of either bronchodilator reversibility testing or metacholine challenge (www.ginasthma.org).

All participants, except for several asthmatic subjects, were non-atopic as determined by negative results of skin prick testing with common aeroallergens. COPD patients were treated with bronchodilators only. Subjects with a history of infection, or who had used antibiotics or systemic/inhaled steroids within the previous four weeks were excluded. All subjects gave their written informed consent, and the study was approved by the Ethics Board of the Medical University of Lodz. The detailed characteristics of the subjects are presented in Table 1 and Table 2. Patients with COPD were evaluated using COPD Assessment Test (CAT), modified Medical Research Council dyspnea scale (mMRC), six-minute walking test (6MWT) and BODE index (see Additional file 1).

**Table 1:** Characteristics of subjects included in the exhaled breath condensate (EBC) study

	Healthy Non-smokers	Asthma	COPD
Number of subjects	16	23	25
Sex (M/F)	11/5	11/12	11/14
Age (years)	56.75 ± 2.71	54.22 ± 3.05	66.96 ± 1.76^ll**^
Pack-years	0	0	39.10±3.70
Time since diagnosis (years)	–	10.13 ± 2.34	4.46 ± 0.84^‡^
PB FEV_1_% of predicted (%)	107.90 ± 3.23	82.59 ± 4.56	62.32 ± 2.76^*†^
PB FEV_1_/FVC (%)	76.32 ± 1.46	68.14 ± 2.38	54.03 ± 1.89^*†^
Current smokers (%)	0	0	32
Use of ICS (%)	0	0	0
Atopic subjects (%)	0	56.52	0
Blood eosinophil number (cells/μl)	181 ± 35	315 ± 35^§^	242 ± 31
Blood eosinophil percentage (%)	3.01 ± 0.54	4.74 ± 0.59^§^	3.16 ± 0.41

*PB* post bronchodilator, *FEV*_*1*_ forced expiratory volume in first second, *FVC* forced vital capacity, *ICS* inhaled corticosteroids

^*^COPD vs Asthma *p* < 0.001; ^†^COPD vs Healthy Non-smokers *p* < 0.001; ^‡^COPD vs Asthma *p* < 0.01; ^§^Asthma vs Healthy Non-smokers *p* < 0.05; ^ll^COPD vs Healthy Non-smokers *p* < 0.05; ^**^COPD vs Asthma *p* < 0.01

**Table 2:** Characteristics of subjects included in the sputum study

	Healthy Non-smokers	Healthy Smokers	COPD
Number of subjects	20	20	40
Sex (M/F)	10/10	11/9	22/18
Age (years)	56.84±2.14	57.85±2.00	67.40±1.12^a†^
Pack-years	0	32.14±2.96	41.99±2.78^†^
Time since diagnosis (years)	–	–	7.05±1.00
PB FEV_1_% of predicted	101.9±2.92	96.88±6.26	61.98±2.76^a†^
PB FEV_1_%FVC	77.69±1.43	74.39±1.18	52.49±1.57^a†^
BMI (kg/m^2^)	27.23 ± 0.80	27.72 ± 0.68	28.39 ± 1.05
CAT score	–	–	15.8 ± 1.18
6MWT (m)	–	–	388.6 ± 11.08
mMRC score^‡^	–	–	1 (0–3)
BODE index^‡^	–	–	1 (0–6)
Current smokers (%)	0	100	42.5
Use of ICS (%)	0	0	0
No. of atopic subjects (No)	0	0	0
Sputum eosinophils (%)	0.89 ± 0.28	1.43 ± 0.28	2.93 ± 0.95
Sputum neutrophils (%)	18.89 ± 3.77	23.28 ± 2.23	37.40 ± 4.93^a^
Sputum macrophages (%)	65.69 ± 3.76	56.85 ± 1.99	44.70 ± 41^a#^
Sputum lymphocytes (%)	6.43 ± 0.84	4.26 ± 0.72	6.60 ± 1.99
Sputum eosinophils (No of cells/ g of sputum × 10^4^)	1.60 ± 0.77	1.55 ± 0.73	2.70 ± 1.33
Sputum neutrophils (No of cells/ g of sputum ×10^4^)	14.95 ± 3.12	19.40 ± 10.01	19.50 ± 7.36
Sputum macrophages (No of cells/ g of sputum ×10^4^)	16.10 ± 2.79	31.00 ± 5.62	41.98 ± 12.55
Sputum lymphocytes (No of cells/ g of sputum ×10^4^)	2.60 ± 0.47	2.10 ± 0.45	5.92 ± 3.25

*PB* post bronchodilator, *FEV*_*1*_ forced expiratory volume in first second, *FVC* forced vital capacity, *CAT* COPD assessment test, *6MWT* six-minute walk test, *mMRC* modified Medical Research Council dyspnea scale, *ICS* inhaled corticosteroids

^a^COPD vs Healthy Non-smokers, *p* < 0.05; ^†^COPD vs Healthy Smokers *p* < 0.05; ^‡^median (min-max)

### Biological samples collection and measurements

The EBC was collected according to the recommendations of the European Respiratory Society [14]. Sputum samples were induced using hypertonic saline and processed as described previously [15]. The COPD subjects were then divided into subgroups based on sputum eosinophil count: one group with airway eosinophilic inflammation (i.e. sputum eosinophil level > 3%) and the other without (i.e. sputum eosinophil level ≤ 3%). The 3% cut-off value for sputum eosinophilia was used based on previous studies on eosinophilic airway inflammation in COPD [16, 17]. The serum and sputum supernatants were tested for IL-33 and sST2 concentrations by means of enzyme-linked immunosorbent assays, while the EBC was tested for IL-33 only.

ST2 mRNA expression in peripheral mononuclear cells (PBMC) and sputum cells was measured using qPCR. Hemopoietic progenitor cells expressing ST2 and intracellular IL-5 were enumerated in blood and induced sputum obtained from COPD subjects with and without airway eosinophilia by means of flow cytometry (see gating strategy in Additional file 2). Detailed information on the study procedures can be found in the Additional file 1.

### Statistical analysis

The results were analyzed using GraphPad Prism 6 (GraphPad Software, San Diego, CA). For clarity, all results are presented as means ± SEMs. The normality of data distribution was tested with the Shapiro-Wilk test. Comparisons between healthy non-smokers, asthmatics and COPD subjects, as well as between healthy non-smokers, healthy smokers and COPD subjects, were analyzed with one-way ANOVA and the post hoc Tukey’s test, or with the Kruskal-Wallis test and the post hoc Dunn’s test, where appropriate.

Differences between COPD subjects with and without eosinophilia were analyzed using the Student’s t-test or U-Mann Whitney test, where appropriate. Correlation analysis was conducted using either Pearson’s or Spearman’s correlation coefficient according to the distribution of variables. Significance was accepted at *P* < 0.05.

## Results

### Exhaled breath condensate study

Absolute blood eosinophil number and percentage were elevated in asthmatics compared to healthy non-smokers (*p* < 0.05). No significant difference in blood eosinophil counts was observed between COPD patients, healthy non-smokers and asthmatic subjects (Table 1).

**Fig. 1:**
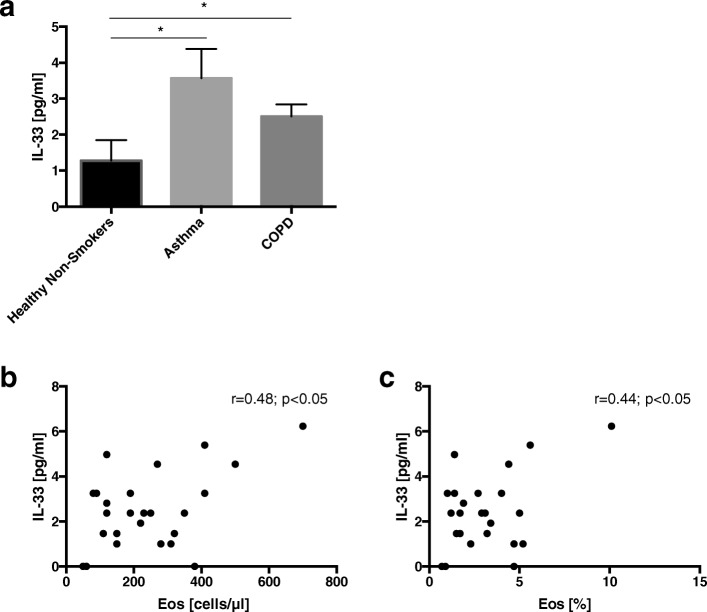
IL-33 concentrations in exhaled breath condensate (EBC) in healthy non-smokers, asthmatics and non-atopic COPD subjects (**a**). Data are presented as mean ± SEM. Correlations between EBC IL-33 and blood eosinophil numbers (**b**) and percentage (**c**) in COPD patients. *p < 0.05

EBC IL-33 levels were significantly higher in asthmatic (3.57 ± 0.81 pg/ml) and COPD (2.50 ± 0.33 pg/ml) patients compared to healthy controls (1.27 ± 0.57 pg/ml), with no significant difference observed between the first two groups (Fig. 1a). IL-33 levels in EBC correlated significantly with blood eosinophil numbers and percentages in the asthmatic (see Additional file 3) and COPD subjects (Fig. 1b and c).

### Sputum study

Sputum differential counts are presented in Table 2. It was found that 25% of COPD subjects had sputum eosinophilia > 3% (mean sputum eosinophil percentage 8.89 ± 3.18%).

**Fig. 2:**
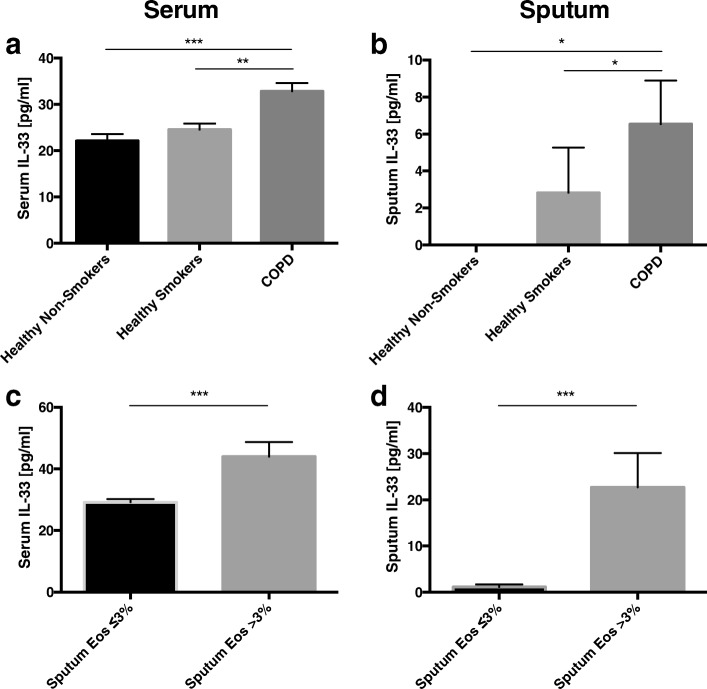
Serum (**a**) and sputum (**b**) IL-33 concentrations in healthy non-smokers, healthy smokers and patients with COPD. Comparison of serum (**c**) and sputum (**d**) IL-33 between COPD subjects with and without sputum eosinophilia. Data are presented as mean ± SEM. *p < 0.05; **p < 0.01; ***p < 0.001

IL-33 serum concentrations were significantly elevated in COPD subjects (32.86±1.74 pg/ml) compared with healthy non-smokers (22.12±1.45 pg/ml; *p* < 0.001) and smokers without COPD (24.45±1.33 pg/ml; *p* < 0.01) (Fig. 2a). In addition, serum IL-33 levels were found to be significantly higher in the subgroup of COPD patients with sputum eosinophilia (43.98±4.76 pg/ml) compared to those with a sputum eosinophil level ≤3% (29.15±2.01 pg/ml; *p* < 0.001) (Fig. 2c).

Sputum IL-33 levels were significantly increased in COPD subjects (6.52 ± 2.37 pg/ml) compared with healthy non-smokers (0.0 ± 0.0 pg/ml; *p* < 0.05) and healthy smokers (2.92 ± 2.45 pg/ml; *p* < 0.05) (Fig. 2b). Similarly to the serum findings, IL-33 sputum levels were significantly higher in COPD subjects with sputum eosinophilia (22.72 ± 7.42 pg/ml) than in those without (1.13 ± 0.56 pg/ml; *p* < 0.001) (Fig. 2d).

**Fig. 3:**
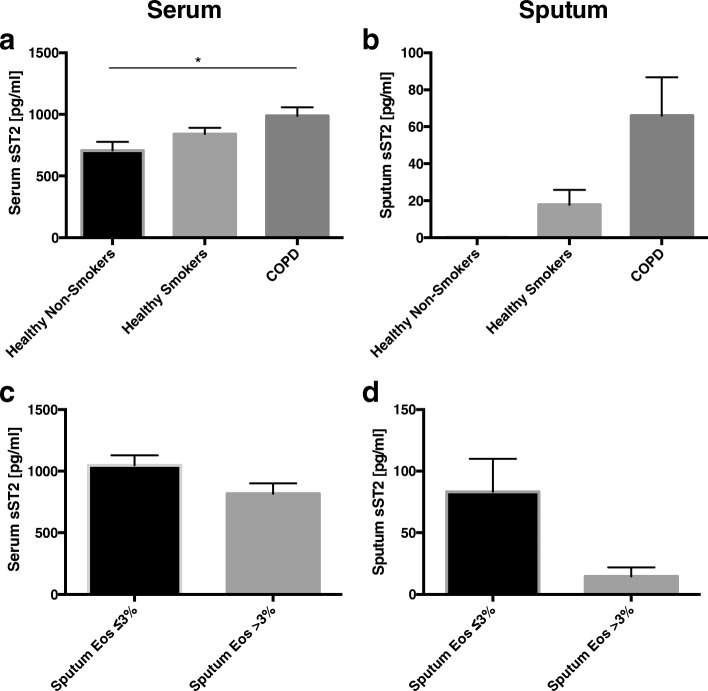
Serum (**a**) and sputum (**b**) sST2 concentrations in healthy non-smokers, healthy smokers and patients with COPD. Comparison of serum (**c**) and sputum (**d**) sST2 between COPD subjects with and without sputum eosinophilia. Data are presented as mean ± SEM. *p < 0.05

sST2 was elevated in the serum (*p* < 0.05) of COPD patients compared with the healthy controls (Fig. 3a). A similar trend was observed for sputum sST2 levels (*p* = 0.059) (Fig. 3b). No significant difference was observed in serum and sputum sST2 levels in COPD subjects with regard to sputum eosinophil counts (Fig. 3c and d).

**Fig. 4:**
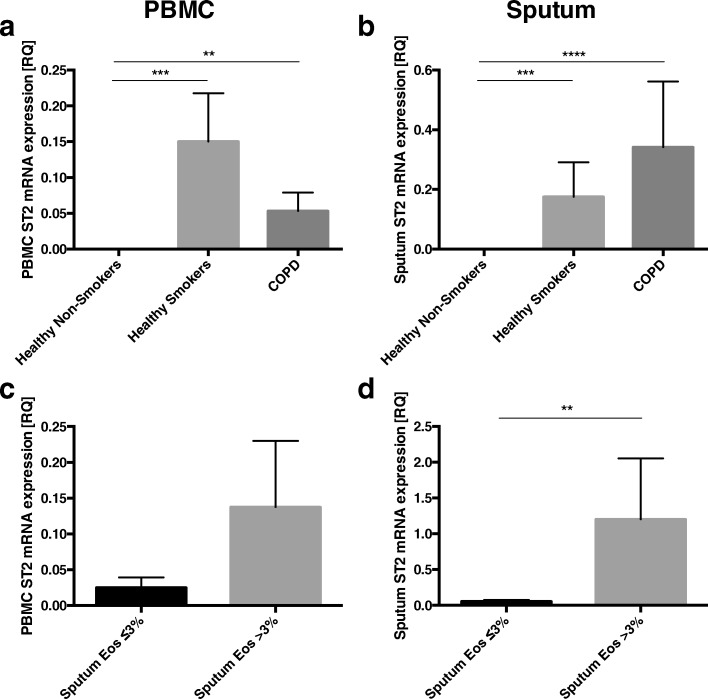
ST2 mRNA expression in peripheral blood mononuclear cells (PBMC) (**a**) and sputum cells (**b**) in n healthy non-smokers, healthy smokers and patients with COPD. Comparison of ST2 mRNA expression in PBMC (**c**) and sputum cells (**d**) between COPD subjects with and without sputum eosinophilia. Data are presented as mean ± SEM. **p < 0.01; ***p < 0.001; ****p < 0.0001

ST2 mRNA was overexpressed in PBMC and sputum cells from healthy smokers and COPD subjects compared with healthy non-smokers (Fig. 4a and b). ST2 mRNA expression was significantly higher in sputum cells from COPD patients with eosinophil airway inflammation (Relative quantification(RQ) = 1.20 ± 0.85) than in those without airway eosinophilia (RQ = 0.05 ± 0.01; *p* < 0.01) (Fig. 4d).

**Fig. 5:**
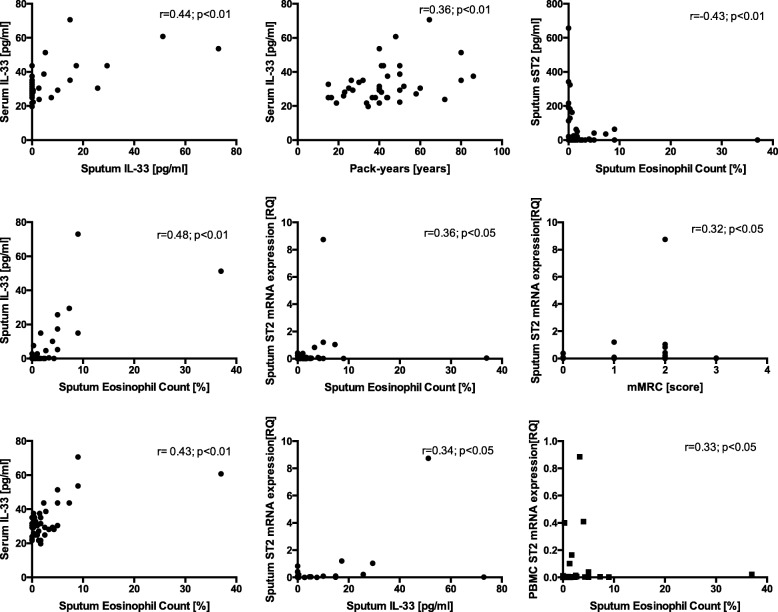
Significant correlations between serum and sputum IL-33 and sST2, ST2 mRNA and clinical parameters in COPD

The significant correlations between IL-33, sST2, ST2 mRNA and sputum eosinophil content and clinical parameters in COPD are presented in Fig. 5. All analyzed correlations are presented in Additional file 4.

**Fig. 6:**
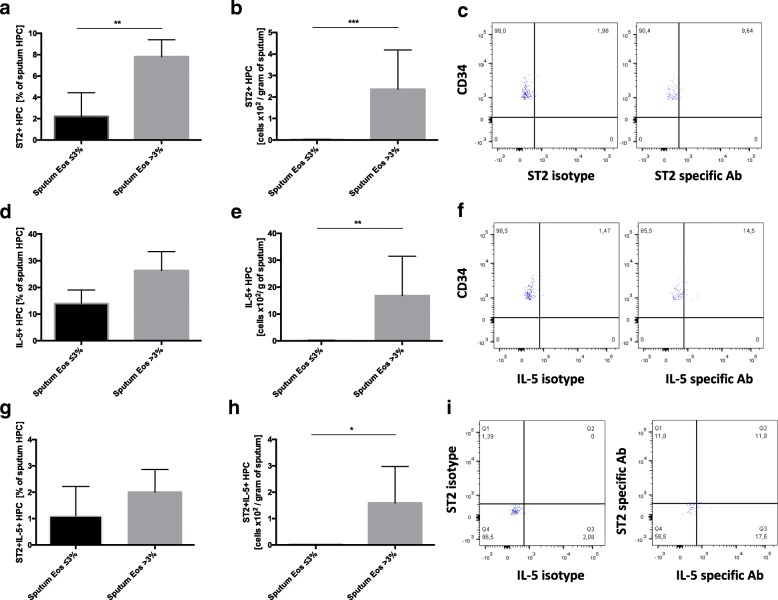
Expression of ST2 and intracellular IL-5 by sputum hemopoietic progenitor cells (HPC) in COPD subjects with sputum eosinophil count ≤3% (*n* = 10) and > 3% (*n* = 10). The percentage of sputum HPC expressing ST2, IL-5 and double positive cells for ST2 and IL-5 are presented in sub-figures **a, d** and **g**, respectively. Absolute numbers of HPC expressing ST2, IL-5 and double positive cells for ST2 and IL-5 per gram of sputum are presented in sub-figures **b**, **e** and **h**, respectively. Representative example of flow cytometry acquisition of sputum ST2+ (**c**), IL-5+ (**f**) and ST2 + IL-5+ (**i**) HPC. Data are presented as mean ± SEM. *p < 0.05; **p < 0.01; ***p < 0.001

No significant difference in circulating HPC count was found between COPD subjects with sputum eosinophilia and those without (422.90 ± 97.65/1 mln PBMC vs 553.70 ± 138.70/1 mln PBMC, respectively; *p* = 0.97). However, COPD subjects with sputum eosinophilia demonstrated significantly higher numbers of sputum HPC than those without eosinophilia (35.64 ± 27.39 × 10^2^ /gram of sputum vs 1.19 ± 0.34 × 10^2^ /gram of sputum, respectively; *p* < 0.001) (see Additional file 5). Similarly, no differences were found between the two groups with regard to percentage or absolute number of circulating ST2 + HPC (see Additional file 6). However, both the percentage of ST2 expression on HPC and the absolute number of ST2 + HPC in sputum were higher in patients with a sputum eosinophil count > 3% compared to those without (7.80 ± 1.58% vs 2.20 ± 2.22%; *p* < 0.01 and 2.36 ± 1.83 × 10^2^ /gram of sputum vs 0.018 ± 0.01 × 10^2^ /gram of sputum; *p* < 0.001, respectively) (Fig. 6a and b).

No differences regarding the percentages and absolute numbers of circulating IL-5 + HPC were observed between COPD patients with sputum eosinophilia and those without (see Additional file 6). In sputum, absolute IL-5 + HPC counts, but not percentages, were elevated in COPD patients with sputum eosinophilia (16.83 ± 14.65 × 10^2^ /gram of sputum vs 0.19 ± 0.07 × 10^2^ /gram of sputum; *p* < 0.01) (Fig. 6d and e).

No difference was observed in circulating ST2 + IL-5+ HPC between the two subgroups of COPD patients (see Additional file 6). However, the absolute numbers of ST2 + IL-5+ HPC, but not their percentage, were significantly higher in the sputum of eosinophilic COPD patients than in those with normal COPD (1.58 ± 1.38 × 10^2^ /gram of sputum vs 0.009 ± 0.009 × 10^2^ /gram of sputum; *p* < 0.001) (Fig. 6g and h).

No correlation was found between the percentages (*r* = 0.24; *p* = 0.28) or absolute numbers (*r* = 0.30; *p* = 0.38) of ST2+ and IL-5+ HPC in sputum.

## Discussion

Our findings show for the first time that increased expression of IL-33 and its receptor ST2 is associated with airway eosinophilia in non-atopic COPD patients.

IL-33 has been extensively studied in allergic asthma and other allergic conditions. Some studies suggest that IL-33 may be also implicated in the pathogenesis of COPD. IL-33 and sST2 serum levels are elevated in COPD patients compared to healthy smokers [18]. Furthermore, immunofluorescence staining showed that the expression of IL-33 was increased in the lung tissues of patients with COPD, indicating that IL-33 upregulation was probably related to systemic and airway inflammation in COPD [18–20]. It has also been reported that cigarette smoke markedly enhanced the expression of IL-33 and ST2 in the lung tissue of mice, which was accompanied by neutrophil and macrophage infiltration and elevated expression of pro-inflammatory cytokines and chemokines in the airways [19]. Interestingly, Kim et al. found blood eosinophil counts to be correlated with plasma IL-33 levels in COPD patients [20].

Recently, Gluck et al. showed that IL-33 protein is detectable at low levels in EBC, and its level is elevated in asthmatics compared with healthy controls [21]. Our results demonstrate that IL-33 is elevated in EBC collected from non-atopic COPD subjects to the same extent as in asthma. Furthermore, EBC IL-33 levels correlated positively with blood eosinophil numbers and percentages, not only in asthma but also in COPD.

To further explore these findings, the second part of our study investigated the expression of IL-33, its receptor and its soluble form in the periphery and the airways of patients with and without airway eosinophilia. Our results showed that the level of IL-33 protein is markedly increased in the serum and sputum of patients with eosinophilic COPD.

sST2 is a decoy receptor regulating the activity of IL-33. As sST2 is not detected in EBC, its levels were not assessed in the EBC obtained from COPD patients [21]. However, our findings confirm those of previous reports showing increased levels of serum sST2 in COPD [18]. These findings also suggest a trend toward overexpression of sputum sST2 in the whole COPD group. Animal studies have shown that sST2 may exert a protective function in acute lung injury and ovalbumin-induced allergic asthma [22, 23], and Hacker et al. suggest that sST2 plays a critical role in the anti-inflammatory regulatory mechanism in early stages of COPD [24]. Although our findings do not indicate significant differences in sST2 concentrations between COPD subjects with and without sputum eosinophilia, sputum sST2 levels nevertheless correlated negatively with sputum eosinophil counts, suggesting it plays a protective role against airway eosinophilic inflammation.

On the contrary, sputum cells obtained from COPD patients with airway eosinophilia overexpressed ST2 mRNA. ST2 is expressed by many types of cells that may be present in the airways, including eosinophils. The activation of eosinophils by IL-33 through ST2 leads to the production of superoxide and IL-8 and increases eosinophil survival [25]. It is important to note that IL-33 activates eosinophils at least to the same degree as IL-5 [25]. The significant correlations between IL-33 and ST2 and sputum eosinophil counts found in our study support the hypothesis that IL-33 may be involved in the development and maintenance of eosinophilic airway inflammation in non-atopic COPD subjects.

Finally, the HPC count as well as ST2 and intracellular IL-5 expression by these cells were measured in the patients with sputum eosinophilia and in those without. HPC are known to act as pro-inflammatory effector cells of allergic inflammation [11]. HPC express ST2 and IL-33 as potent activators of HPC, leading to the release of a number of cytokines and chemokines, including IL-5. HPC can differentiate into mature eosinophils at the site of inflammation in a process called “*in situ* eosinophilopoiesis”. IL-33 accelerates the maturation of HPC and modulates their migration into airways in allergic asthma [26, 27].

Little is known of the role of HPC in COPD. Some studies have reported reduced numbers of circulating HPC in COPD [28], while others report similar circulating and sputum HPC numbers between COPD and healthy non-atopic subjects [29]. Our present findings do not suggest any differences in circulating HPC numbers between COPD patients with sputum eosinophilia and those without. However, a striking difference was observed in the number of sputum HPC between the two groups of COPD patients, with significantly elevated HPC numbers found in those with sputum eosinophils > 3%. This was accompanied by overexpression of intracellular IL-5 and ST2 by sputum HPC indicating increased activation of these cells in eosinophilic COPD, analogously to allergic asthma.

As IL-33 modulates the trafficking of HPC, it is possible that increased IL-33 levels may be at least partially responsible for the augmented influx of HPC into airways observed in COPD patients with eosinophilic inflammation. In addition, increased numbers of ST2 + IL-5 + HPC were seen in the sputum of patients with airway eosinophilia. This finding suggests that IL-33 activates HPC in eosinophilic COPD. Therefore, in those subjects, HPC may act as effector cells in an analogous way to allergic asthma, by fostering the development of a local IL-5 rich environment independent of the IgE pathway.

There are several limitations to our study. First, the IL-33 protein levels were low in a significant number of exhaled breath and sputum specimens. This could be due to the rapid neutralization of IL-33 following its release from activated cells. Measuring IL-33 protein content is challenging and previous studies give varying results for serum and sputum [30, 31]. Nonetheless, our findings on ST2 expression confirm the IL-33 measurements and support the association between IL-33 and eosinophilic phenotype of COPD. The best way to determine IL-33 expression would be to measure it directly in the main source of the cytokine, i.e. the airway epithelium; however, studies comparing IL-33 expression in eosinophilic COPD involving invasive methods are warranted. In addition, the results may have been affected by the fact that our group of COPD subjects was older than those of the other two groups. However, as no correlation has been found between IL-33 and ST2 expression and the age of participant, it is unlikely that this may be the case.

## Conclusions

In conclusion, our results suggest that increased IL-33 is associated with airway eosinophilia in non-atopic COPD. It is tempting to speculate that IL-33 is involved in the recruitment and activation of HPC into the airways. This may result in the creation of a local, IL-5 rich inflammatory state similar to that observed in allergic asthma. Thus, IL-33 may be a potential therapeutic target in the subgroup of COPD patients characterized by eosinophilic inflammation.

## Additional files

Additional file 1: Detailed Method description. (PDF 118 kb)

Additional file 2: **Figure E4.** Hemopoietic progenitor cells gating strategy. (PDF 88 kb)

Additional file 3: **Figure E1.** Correlations between IL-33 concentrations in exhaled breath condensate and blood eosinophil numbers (A) and percentage (B) in asthmatic patients. (PDF 49 kb)

Additional file 4: **Table E1.** Correlations between serum and sputum IL-33 and sST2, ST2 mRNA and clinical parameters in COPD. FEV1 – forced expiratory volume in first second; FVC – forced vital capacity; CAT – COPD assessment test; 6MWT – six-minute walk test; mMRC – modified Medical Research Council dyspnea scale. (PDF 39 kb)

Additional file 5: **Figure E2.** Circulating (A) and sputum (B) hemopoietic progenitor cells (HPC) in patients with and without sputum eosinophilia. **p < 0.01. (PDF 56 kb)

Additional file 6: **Figure E3.** The percentage and absolute numbers of circulating hemopoietic progenitor cells (HPC) expressing ST2 (A and B, respectively), intracellular IL-5 (C and D, respectively) and double positive for ST2 and IL-5 (E and F, respectively) in COPD patients with (sputum eosinophils > 3%) and without (sputum eosinophils ≤3%) sputum eosinophilia. (PDF 51 kb)

## Abbreviations

6MWT: Six-minute walking test
CAT: COPD Assessment Test
COPD: Chronic Obstructive Pulmonary Disease
EBC: Exhaled breath condensate
GINA: Global Initiative for Asthma
GOLD: Global Initiative for Chronic Obstructive Lung Disease
HPC: Hemopoietic progenitor cells
IL: Interleukin
mMRC: Modified Medical Research Council
PBMC: Peripheral mononuclear cells
RQ: Relative quantification
sST2: Soluble ST2
ST2: IL-33 receptor
