# Supplementary material for: The association between airway eosinophilic inflammation and IL-33 in stable non-atopic COPD
Source: Respir Res. 2018 Jun 1;19:108. doi: 10.1186/s12931-018-0807-y (PMC5984757; doi:10.1186/s12931-018-0807-y)
Supplement: Supplementary file 1 — Detailed Method description. (PDF 118 kb) [file 12931_2018_807_MOESM1_ESM.pdf]

Additional File 1 – Detailed Method Description

The Association Between Airway Eosinophilic Inflammation and IL-33 in Stable Non-Atopic COPD

Damian Tworek, Sebastian Majewski, Karolina Szewczyk, Justyna Kiszałkiewicz, Zofia Kurmanowska, Paweł Górski, Ewa Brzeziańska-Lasota, Piotr Kuna, Adam Antczak

Methods:

The COPD assessment test (CAT):

CAT is a simple, validated health status instrument for patients with COPD. The self-administered questionnaire consists of eight items assessing various manifestations of COPD and global impact of the disease on health status. It is a simple, quantified measure of health-related quality of life. CAT scores range from 0 to 40. A decrease in CAT score represents an improvement in health status, whereas an increase in CAT score represents a worsening in health status (1).

Six-minute walk test (6MWT):

The 6MWT is used for the evaluation of functional exercise capacity in patients with chronic respiratory diseases. 6MWT will be performed using the methodology specified by the Polish Respiratory Society guidelines (2). Briefly, all COPD patients will be instructed to walk as far as possible for 6 minutes. The 6MWT will be performed in a flat, long, covered, 30 m-long corridor with the metres marked. When the test is finished, the distance covered will be calculated.

The modified Medical Research Council (mMRC) dyspnea scale:

mMRC is a five-level rating scale based on the patient's perception of dyspnea in daily activities. It consists of five statements that describe the entire range of dyspnea from none (Grade 0) to almost complete incapacity (Grade 4) (3).

BODE index:

The BODE index is a multidimensional scoring system for COPD patients which evaluates body mass index (BMI), measure of airflow obstruction (FEV1% predicted), dyspnea score (grade in mMRC scale), and exercise capacity (distance covered in 6MWT). This composite marker of disease takes into consideration the systemic nature of COPD and is used to predict long-term outcomes in this population (4).

Skin prick testing:

All patients underwent skin prick tests performed with common aeroallergens: Dermatophagoides pteronyssinus, Dermatophagoides farinae, grasses, birch, hazel, alder, mugwort, cat, dog, Alternaria tenuis, and Cladosporium herbarum (Allergopharma). Histamine 1.7 mg/mL (Allergopharma) and standard glycerol saline solution (Allergopharma) were used as a positive and negative control, respectively. A wheal diameter ≥3mm was considered a positive result.

Exhaled breath condensate (EBC):

The EBC was collected using a commercial condenser (Thermo Haake EK20, Ecoscreen, Jaeger) according to the recommendations of the European Respiratory Society (5). Patients were asked to breath out spontaneously for 10 min through a mouthpiece equipped with a saliva trap. The respiratory rate ranged from 15 to 20 breaths/min. All subjects wore a nose

clip and rinsed their mouths with distilled water just before and in the seventh minute of collection in order to reduce nasal contamination.

Collected condensate was immediately frozen in -80ºC until ELISA measurements.

Sputum induction and processing:

Sputum samples were induced using hypertonic saline. Selected mucous plugs were processed using a two-step method with a Dulbecco's phosphate-buffered saline (D-PBS) wash step followed by a dithiothreitol (DTT) step and cytospins (6). Sputum supernatants were frozen at −80 °C. Cytospins were prepared from sputum cells and stained with Diff-Quik for differential cell counts. Remaining cells were subjected to measurements of ST2 mRNA expression and flow cytometry staining.

Blood samples processing:

Peripheral venous blood samples were withdrawn into lithium heparin tubes (BD Dickenson) at baseline and at 24 h after the diluent/allergen inhalation challenge. This blood was diluted with McCoys 5A (Invitrogen) and then layered on Lymphoprep (d = 1.077 mg/ml; Axis-Shield) and centrifuged at 2,200 rpm for 20 min at room temperature. Peripheral blood mononuclear cells (PBMC) were removed and washed with McCoy 5A (centrifugation at 1500 rpm for 10 minutes at 4°C). Two million of PBMC per tube were used for staining for flow cytometry. Remaining cells were kept in −80 °C until mRNA expression analysis.

ELISA:

IL-33 and sST2 concentrations in EBC (IL-33 only), serum and sputum supernatants were measured in duplicate using commercially-available enzyme-linked immunosorbent assays (R&D) according to manufacturer’s instructions. In The sensitivity of the assay was 1.65

pg/ml for IL-33 and 13.5 pg/ml for sST2. In the case of values lower than the method sensitivity limit, the samples were quantified based on extrapolation of standard curves generated for each set of samples assayed.

Gene expression analysis in PBMC and sputum cells:

PBMC and sputum cells were resuspended in RNAlater (Thermofisher) and kept in −80 °C until RNA isolation. RNA isolation was performed using the mirVana™ miRNA Isolation Kit (Life Technologies), according to the manufacturer's protocol. The quality and quantity of isolated RNA was spectrophotometrically assessed (Eppendorf BioPhotometrTM Plus). The purity of total RNA (ratio of 16S to 18S fraction) was determined in the automated electrophoresis using the RNA Nano Chips LabChipplates in Agilent 2100 Bioanalyzer (Agilent Technologies).

Complementary DNA (cDNA) was transcribed from 100 ng of total RNA, using a High-Capacity cDNA Reverse Transcription Kit (Applied Biosystems) in a total volume of 20 µl, according to manufacturer's protocol. The relative expression analysis was performed in 7900HT Fast Real-Time PCR System (Applied Biosystems) using TaqMan probes for the following genes: ST2 (Hs00249384_m1) and ACTB (Hs99999903_m1) used as an endogenous control. The PCR mixture contained cDNA (1 to 100 ng), 20× TaqManR Gene Expression Assay, 2× KAPA PROBE Master Mix (2×) ABI Prism Kit (Kapa Biosystems), and RNase-free water in a total volume of 20 µl. The expression levels (RQ values) of the studied genes were calculated using the delta delta CT method, with the adjustment to the β-actin expression level and in relation to the expression level of calibrator (Human Lung Total RNA Ambion®), for which RQ value was equal to 1.

Flow cytometry:

PBMC and sputum cells for flow cytometry experiments were immunostained with isotype or specific mAbs to the extracellular CD45 (Ebioscience, CA, US), CD34 (Ebioscience) and ST2 (R&D). To measure intracellular IL-5 expression, cells were washed, fixed and permeabilized, then stained with isotype control or antibody to IL-5 (R&D). Cells were washed and acquired with a Canto flow cytometer (BD Biosciences). Analyses were performed using Flow-Jo software (Tree Star). HPC were defined as $FSC^{low}SSC^{low}CD45^{dim}CD34^{high}$ population according to the previously described gating strategy (Figure E4)(7) . The isotype control for the markers of interest was set to 2 %, which was compared to the specific markers to detect the percentage of cells expressing the marker.

References:

1. Jones PW, Harding G, Berry P, Wiklund I, Chen W-H, Kline Leidy N. Development and first validation of the COPD Assessment Test. *Eur Respir J* 2009;34:648–54.
2. Przybyłowski T, Tomalak W, Siergiejko Z, Jastrzębski D, Maskey-Warzęchowska M, Piorunek T, Wojda E, Boros P. Polish Respiratory Society guidelines for the methodology and interpretation of the 6 minute walk test (6MWT). *Pneumonol Alergol Pol* 2015;83:283–297.
3. Mahler DA, Wells CK. Evaluation of clinical methods for rating dyspnea. *Chest* 1988;93:580–6.
4. Cote CG, Celli BR. BODE index: a new tool to stage and monitor progression of chronic obstructive pulmonary disease. *Pneumonol Alergol Pol* 2009;77:305–13.
5. Horváth I, Hunt J, Barnes PJ, Alving K, Antczak A, Baraldi E, Becher G, van Beurden WJC, Corradi M, Dekhuijzen R, Dweik RA, Dwyer T, Effros R, Erzurum S, Gaston B, Gessner C, Greening A, Ho LP, Hohlfeld J, Jöbsis Q, Laskowski D, Loukides S, Marlin D, Montuschi P, Olin AC, Redington AE, Reinhold P, van Rensen ELJ, Rubinstein I, *et al.* Exhaled breath condensate: methodological recommendations and unresolved questions. *Eur Respir J* 2005;26:523–48.
6. Bafadhel M, McCormick M, Saha S, McKenna S, Shelley M, Hargadon B, Mistry V, Reid C, Parker D, Dodson P, Jenkins M, Lloyd A, Rugman P, Newbold P, Brightling CE. Profiling of sputum inflammatory mediators in asthma and chronic obstructive pulmonary disease. *Respiration* 2012;83:36–44.
7. Sehmi R, Howie K, Sutherland DR, Schragge W, O'Byrne PM, Denburg JA. Increased levels of CD34+ hemopoietic progenitor cells in atopic subjects. *Am J Respir Cell Mol Biol* 1996;15:645–55.
