# Supplementary figures and images for: The association between airway eosinophilic inflammation and IL-33 in stable non-atopic COPD
Source: Respir Res. 2018 Jun 1;19:108. doi: 10.1186/s12931-018-0807-y (PMC5984757; doi:10.1186/s12931-018-0807-y)

Figure E4.

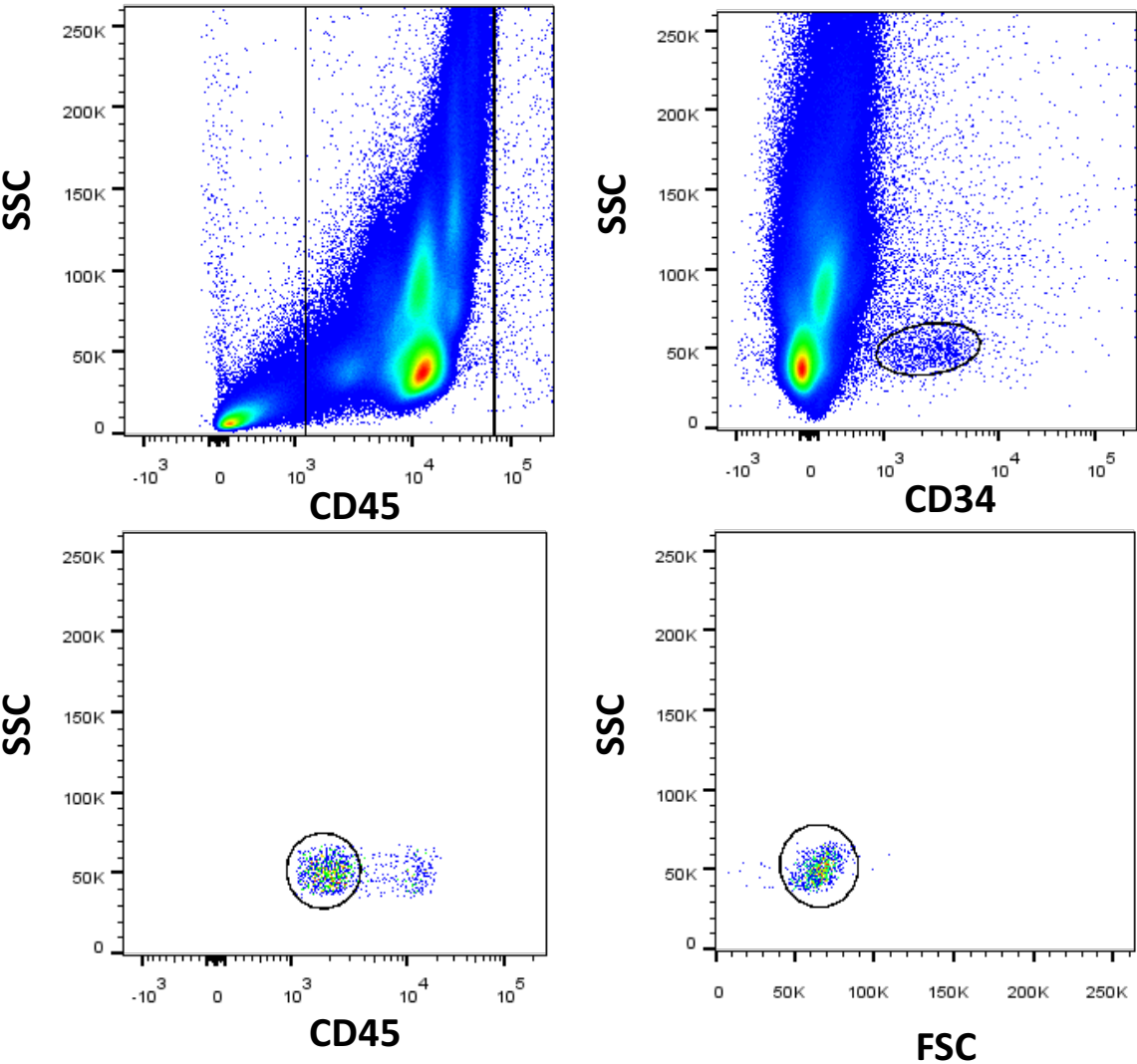

Supplement: Supplementary file 2 — Figure E4. Hemopoietic progenitor cells gating strategy. (PDF 88 kb) [file 12931_2018_807_MOESM2_ESM.pdf]

Figure E1.

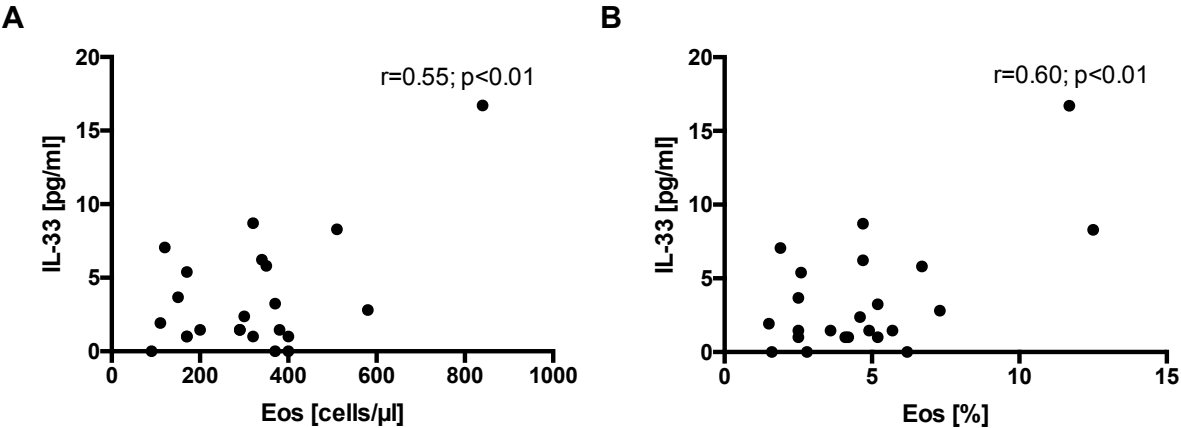

Supplement: Supplementary file 3 — Figure E1. Correlations between IL-33 concentrations in exhaled breath condensate and blood eosinophil numbers (A) and percentage (B) in asthmatic patients. (PDF 49 kb) [file 12931_2018_807_MOESM3_ESM.pdf]

Figure E2.

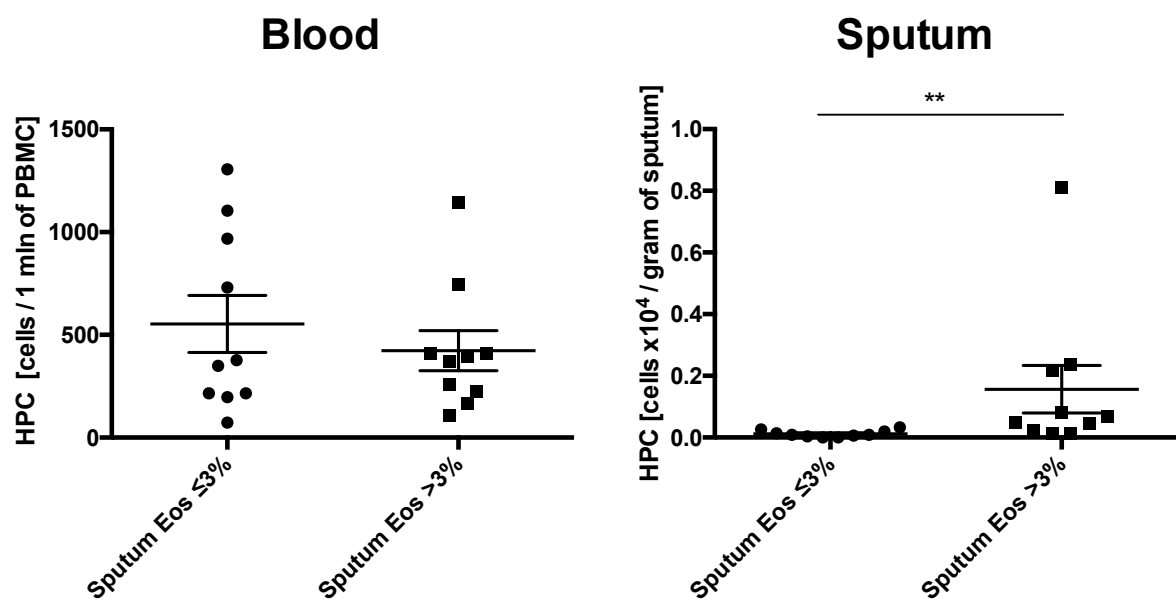

Supplement: Supplementary file 5 — Figure E2. Circulating (A) and sputum (B) hemopoietic progenitor cells (HPC) in patients with and without sputum eosinophilia. **p < 0.01. (PDF 56 kb) [file 12931_2018_807_MOESM5_ESM.pdf]

Figure E3.

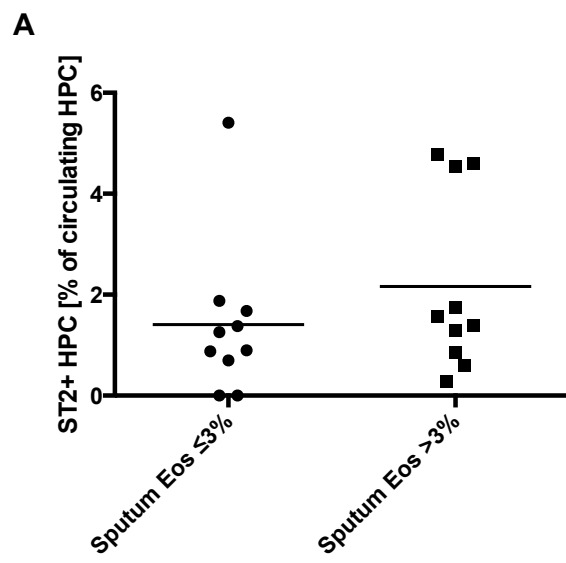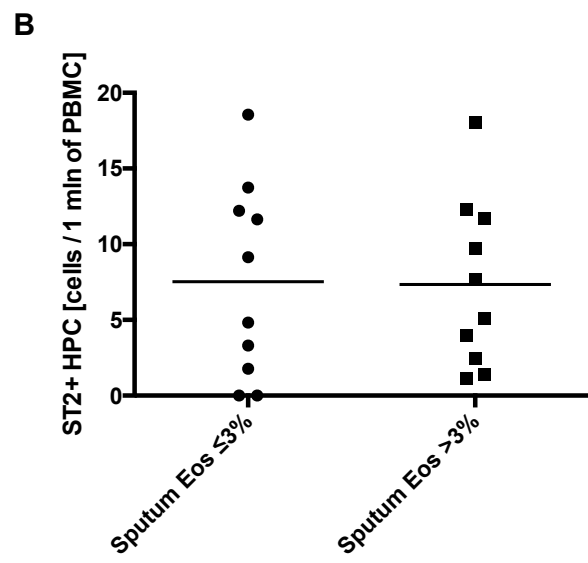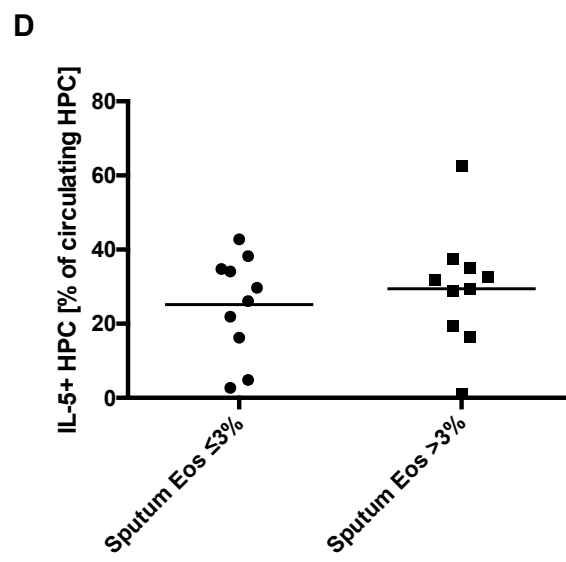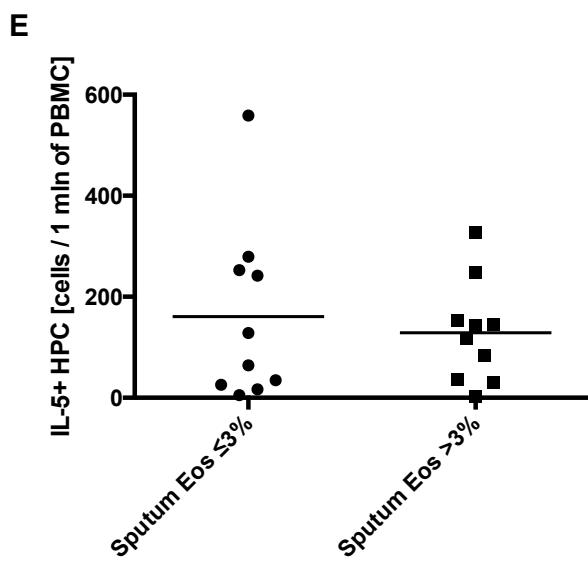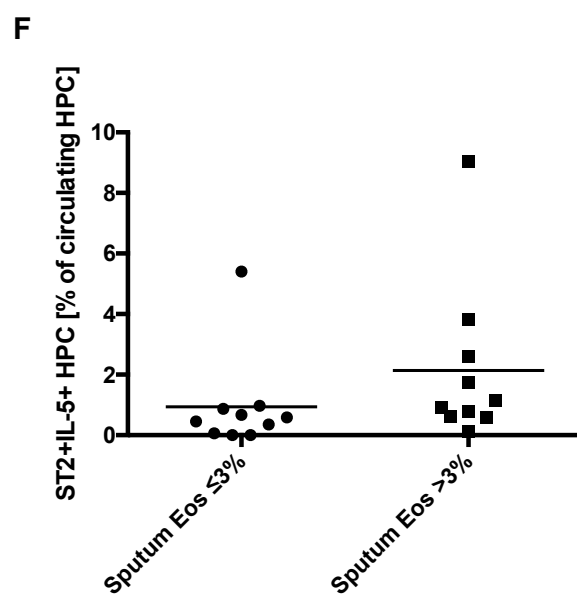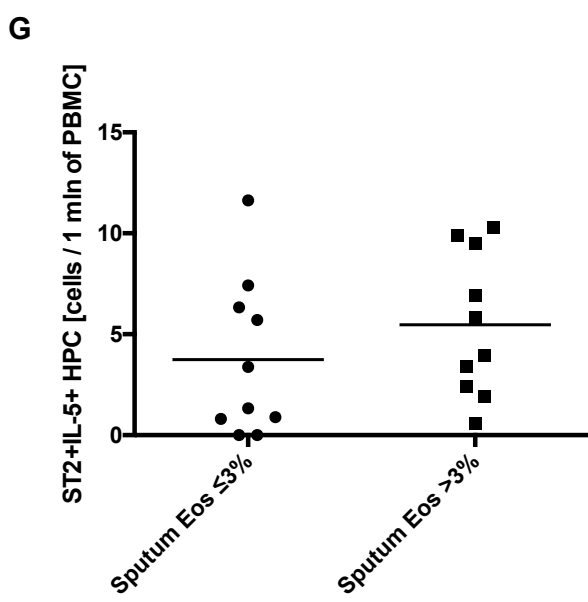

Supplement: Supplementary file 6 — Figure E3. The percentage and absolute numbers of circulating hemopoietic progenitor cells (HPC) expressing ST2 (A and B, respectively), intracellular IL-5 (C and D, respectively) and double positive for ST2 and IL-5 (E and F, respectively) in COPD patients with (sputum eosinophils > 3%) and without (sputum eosinophils ≤3%) sputum eosinophilia. (PDF 51 kb) [file 12931_2018_807_MOESM6_ESM.pdf]
