# Supplementary material for: The association between airway eosinophilic inflammation and IL-33 in stable non-atopic COPD
Source: Respir Res. 2018 Jun 1;19:108. doi: 10.1186/s12931-018-0807-y (PMC5984757; doi:10.1186/s12931-018-0807-y)
Supplement: Supplementary file 4 — Table E1. Correlations between serum and sputum IL-33 and sST2, ST2 mRNA and clinical parameters in COPD. FEV1 – forced expiratory volume in first second; FVC – forced vital capacity; CAT – COPD assessment test; 6MWT – six-minute walk test; mMRC – modified Medical Research Council dyspnea scale. (PDF 39 kb) [file 12931_2018_807_MOESM4_ESM.pdf]

Table E1

	Age	Time since diagnosis	Pack-years	FEV1% of predicted	FEV1/FVC	CAT	mMRC	6MWT	BODE	% of Sputum eosinophils
Serum IL-33	R=-0.13; p=0.41	R=-0.05; p=0.71	R=0.36; p<0.05	R=0.04; p=0.80	R=0.26; r=0.10	R=-0.04; p=0.77	R=0.11; p=0.48	R=-0.26; p=0.10	R=0.21; p=0.17	R=0.43; p<0.01
Sputum IL-33	R=-0.00; p=0.97	R=-0.00; p=0.95	R=0.16; p=0.31	R=-0.18; p=0.26	R=0.05; p=0.74	R=0.19; p=0.23	R=0.01; p=0.91	R=-0.01; p=0.90	R=0.23; p=0.15	R=0.48; p<0.01
Serum sST2	R=-0.21; p=0.17	R=0.20; p=0.20	R=-0.04; p=0.79	R=-0.21; p=0.19	R=-0.13; p=0.40	R=0.31; p=0.05	R=0.12; r=0.42	R=-0.09; p=0.56	R=0.24; p=0.13	R= 0.11; p=0.48
Sputum sST2	R=0.06; p=0.69	R=-0.00; p=0.95	R=0.26; p=0.10	R=-0.14; p=0.38	R=-0.14; p=0.37	R=0.13; p=0.40	R=-0.07; p=0.66	R=0.03; p=0.83	R=0.02; p=0.87	R=-0.43; p<0.01
PBMC ST2 mRNA	R=0.03; p=0.21	R=-0.08; p=0.62	R=0.05; p=0.71	R=-0.03; p=0.84	R=-0.00; p=0.96	R=0.14; p=0.38	R=0.26; p=0.10	R=0.04; p=0.78	R=0.08; p=0.62	R=0.033; p<0.05
Sputum ST2 mRNA	R=-0.00; p=0.97	R=-0.25; p=0.12	R=-0.19; p=0.24	R=-0.11; p=0.46	R=-0.27; p=0.09	R=-0.09; p=0.56	R=0.32; p<0.05	R=0.09; p=0.56	R=0.19; p=0.21	R= 0.36; p=0.02
